# Towards virtual training of emotion regulation

**DOI:** 10.1007/s40708-014-0004-9

**Published:** 2014-09-26

**Authors:** Tibor Bosse, Charlotte Gerritsen, Jeroen de Man, Jan Treur

**Affiliations:** 1 Agent Systems Research Group, Vrije Universiteit Amsterdam, De Boelelaan 1081, 1081 HV Amsterdam, The Netherlands; 2 Netherlands Institute for the Study of Crime and Law Enforcement, De Boelelaan 1077a, 1081 HV Amsterdam, The Netherlands

**Keywords:** Virtual training, Emotion regulation, Emotional response

## Abstract

For professionals in military and law enforcement domains, learning to regulate one’s emotions under threatening circumstances is crucial. The STRESS project envisions a virtual reality-based system to enable such professionals to train their emotion regulation skills. To explore the possibilities for such a system, this article describes an experiment performed to investigate the impact of virtual training on participants’ experienced emotional responses in threatening situations. A set of 15 participants were asked to rate the subjective emotional intensity of a set of affective pictures at two different time points, separated by 6 h. The participants were divided into three groups: the first group performed a session of virtual training in between, in which they received a choice-reaction task; the second group performed a session of virtual training, in which they had to apply reappraisal strategies; and a control group, that did not have any training session. The results indicate that the reappraisal-based training caused the participants in that group to give significantly lower ratings for the emotional intensity of the negative pictures, whereas the content-based training resulted in significantly higher ratings compared to the group without training. Moreover, a second experiment, performed with the same participants 6 months later, indicated that these effects are fairly persistent over time, and that they transfer to different pictures with similar characteristics.

## Introduction

The ability to cope with negative stimuli from the environment is a useful characteristic of human beings. Almost on a daily basis, we are confronted with situations that in one way or the other invoke negative emotions. A particular type of negative emotion, which is typically induced by perceived threats, is *fear* [3]. Depending on the person, different types of stimuli that may trigger fear vary from horror movies and scary animals to enclosed spaces and public speaking. The probability of being confronted with such stimuli depends, among others, on the person’s profession. On average, professionals in domains such as the police, military and public transport are more likely to be confronted with fear-inducing stimuli than people with an office job. It is therefore not surprising that these types of job are usually more appropriate for people who are strong at regulating their levels of fear.

Nevertheless, even the ‘coolest’ of individuals may have difficulties to function adequately in case the stimuli are extreme, such as in cases of military missions or terrorist attacks. First, the extreme emotions experienced in these situations may impair their cognitive processes like attention and decision making [4, 5]. And second, even if they make optimal decisions from an external perspective, they have an increased risk of developing anxiety related disorders such as post-traumatic stress disorder (PTSD) [6]. For these reasons, much time and money is spent on developing appropriate training in these domains. Increasingly, often, virtual environments are successfully used to train performance and decision making of professionals under more realistic and stressful situations (see for example [7] or [8]). Furthermore, methods to prevent or treat PTSD after a traumatic event are costly and may even have negative effects [9]. *Primary prevention*, before any traumatic event has occurred, has been proposed as a promising alternative [10, 11]. A promising technique for primary prevention, which has recently received much attention, is ‘stress inoculation training’ based on virtual reality (VR). The assumption behind this approach is that, by gradually exposing a trainee to fear-provoking stimuli, a VR system is able to increase her ‘mental readiness’ [12, 13]. In that sense, this approach has similarities with exposure therapy [14, 15]. VR-based stress training has proved to be successful, among others, for bank employees [16] and airline crew [17], to increase preparation for hostage situations.

The research presented in this article is part of a large project called STRESS, which stands for simulation-based training of resilience in emergencies and stressful situations (http://stress.few.vu.nl). The main aim of the project as a whole is to develop an intelligent system that is able to analyse human emotion regulation and decision making processes in threatening circumstances, and analyse the causes of incorrect decisions and inadequate stress regulation. The system will be incorporated in an electronic training environment for employees in the public domain, based on VR, cf. [18]. In this environment, trainees will be placed in a virtual scenario, in which they have to make difficult decisions, while negative emotions are induced. During the scenario, modern human computer interaction (HCI) techniques will be applied to measure their emotional state. This information will then be used as input for the intelligent system, to determine why the trainee made certain less optimal decisions and to teach her how to improve this.

The current article focuses on three questions that are relevant for the development of this training environment:

1. What type of training should be provided in order to maximise training effectiveness in reducing negative emotional effects?
2. What are the long-term effects of such types of training?
3. To what extent is there transfer of training to different, but comparable stimuli?

The article makes some steps towards the investigation of these research questions by means of an experiment where participants were exposed to negative stimuli via a computer screen. The article is organised as follows. In Sect. 2, some theoretical background of the research is reviewed. Next, in Sect. 3, an experiment is introduced that was used to assess the impact of different types of virtual training on the experienced emotional intensity towards the stimuli presented. The experiment involves a first part that was mainly designed to investigate research question (1), and a second part (performed after 6 months) to address research question (2) and (3). In Sect. 4, the results of the experiment are presented, and Sect. 5 concludes the article with a discussion.

## Theoretical background

Driven by the goal to develop a virtual environment to train mental readiness, it should be possible to obtain a learned effect of successfully lowering subjects’ stress responses for future situations in such an environment. Type of instructions given to the trainees, in order to obtain a successful learning process, are of great importance. Previous research outside the VR domain (e.g., [19]) suggests that the effectiveness of exposure therapy is partly determined by the specific type of therapy and the task instructions (e.g., related to emotion regulation) that are prescribed to the participant. To investigate the possibilities, we conducted an experiment in which participants’ reactions to viewing negative pictures from the International Affective Picture System (IAPS) picture set [20] were assessed, and the impact of performing reappraisal-based training in a VR environment was studied.

**Fig. 1:**
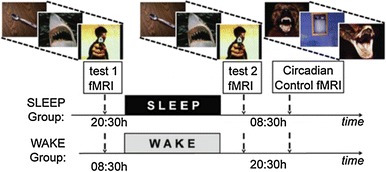
Experimental design used in [21]

The setup of this experiment, which is described in the next section, was inspired by an experiment by Helm et al. in the context of rapid eye movement (REM) sleep [21]. According to recent cognitive and neurological literature, there are striking similarities between virtual exposure therapy and dreaming. In particular, the process of dreaming makes use of memory elements and their associated emotions to generate ‘virtual simulations’, and strengthening of regulation of negative emotions is considered an important purpose of dreaming [22]. For this reason, the idea was to test whether virtual training has a similar effect on emotional responses as is reported in Helm et al.’s experiments [21] about REM sleep. More specifically, in that paper, the experimental design displayed in Fig. 1 was used. The authors summarise the experiment as follows:

34 healthy adults (age: 18–30 years) were randomly assigned to one of two groups. Each performed two repeat fMRI tests (test 1, test 2), separated by 12 h containing a night of EEG-recorded sleep (sleep group, *n* = 18, ten females) or a waking day (wake group, *n* = 16, nine females). During each test, participants viewed and rated the subjective emotional intensity of 150 standardised affective pictures on a 1–5 scale, corresponding to increasing intensity. Importantly, participants viewed the same stimuli at both test sessions, affording a measure of change in emotional reactivity to previously experienced affective stimuli (test2–test1), following wake or sleep. Participants additionally performed a circadian control test at the second fMRI session, involving presentation of a novel set of affective stimuli. This control test allowed confirmation that behavioural and fMRI differences in reactivity identified following wake and sleep were independent of time of day. [21]

**Fig. 2:**
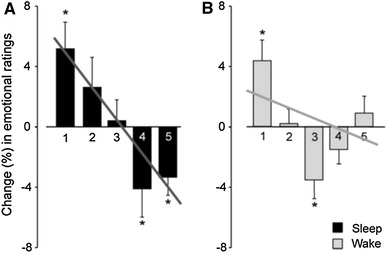
Relative change in emotional ratings depicted in [21]

The results of this experiment showed, among others, that the decrease (between the first and the second test) in subjective emotional ratings of the affective pictures was significantly lower in the sleep group than in the wake group. More specifically, Fig. 2 shows for both the sleep group and the wake group, the relative change in emotional ratings between the first and the second test. For example, the sleep group gave almost 4 % fewer 5-ratings (i.e., the highest subjective ratings for emotional intensity) in the second test, whereas the wake group gave a bit more 5-ratings.

In the next section, our variant of this experiment is described, in which the difference between sleep and wake is replaced by the difference between virtual training and no virtual training.

## Experimental design

The first research question addressed is what type of VR-based training is appropriate, in order to obtain a successful decrease of emotional responses towards negative stimuli. As mentioned above, the effectiveness of exposure therapy is partly determined by the way the person deals with the negative stimuli. Within the context of virtual training, a number of strategies can be used, varying from just looking at the stimuli to performing different emotion regulation strategies such as ‘attentional deployment’ (i.e., directing one’s attention away from the emotional stimulus), ‘cognitive change’ (i.e., changing how one appraises a stimulus so as to alter its emotional meaning), and ‘suppression’ (i.e., inhibiting one’s emotional expression) [23]. As a first step to investigate and compare the effects of different strategies, an experiment was performed in which participants’ reactions to viewing negative pictures from the IAPS picture set, ‘developed to provide a set of normative emotional stimuli’ [20], were assessed for two different types of training. The setup of this experiment is described in the current section.

### Participants

Fifteen healthy adults (of age between 26 and 32 years, with a mean of 28.2) participated in the experiment, and were randomly assigned to one of three groups (to which we will refer as the ‘training 1’ group, the ‘training 2’ group and the ‘no training’ group), in such a way that each group consisted of 5 participants. Six of the participants were female and nine were male.

### Setup

**Fig. 3:**
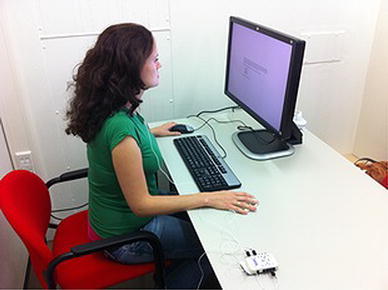
Experimental setup

In the first part of the experiment, the participants in the control (no training) group participated in two rounds, separated by a pause of 6 h (see Fig. 3, lowest line, test 1 and 2). In these rounds they were presented 5 times 30 pictures from the IAPS picture set [20]. The sets of pictures used were identical to the sets used by Helm et al. [21], covering pictures with valence scores ranging from 1.45 (negative) to 8.28 (positive) and arousal scores from 2.28 (low) to 7.12 (high), according to the standard IAPS classification (ranging from 1 to 9). The participants were first shown a black fixation mark for 500 ms, after which the image was shown for 2000 ms. For 2500 ms after the image, a question was shown asking the participants to rate the emotional intensity of the picture on a scale from 1 to 5 (1 being non emotional and 5 being very emotional).^1^ Finally, for another 2700 ms a grey fixation mark was shown, followed by the same sequence for the next image, and so on. The images were shown in 5 blocks of 30 images with small breaks (of about 45 s) in between. Image order was fixed for each block of images, but the order of blocks was randomised between participants. For a total of 150 images, the participants needed approximately 20–25 min to complete the test. Furthermore, the heart rate and the skin conductance of the participants were measured with the PLUX wireless sensor device (http://www.plux.info/).^2^ See Fig. 3 for a picture of the experimental setup.

The participants in both training groups also participated in these rounds, just like the control group. However, in between these two rounds they performed a virtual training session. This training occurred 3 h after the first round and 3 h before the second round (see Fig. 1, upper and middle line). The virtual training made use of the same pictures used in the other rounds. Within the training 1 group, the participants were given a choice-reaction task in which they had to assess the valence of the picture as quickly as possible while the image increased in size up to double the size of the original picture (i.e., whether it gave them a positive or a negative emotion). They could make this distinction by either clicking the mouse or pressing the spacebar. Within the training 2 group, the participants were asked to view them while actively reducing their emotional response until they felt comfortable looking at the picture (e.g., by assuring themselves that the pictures were not real). The motivation for using these two types of training is that we wanted to investigate the impact of type of training and task instructions (i.e., learning to work with potentially negative stimuli in a dynamic context versus learning to cope with negative stimuli directly by performing reappraisal) on subjective emotional response. Although a large number of emotion regulation strategies exist, we selected reappraisal because this strategy has proven successful in a number of related domains (e.g. [24]).

**Fig. 4:**
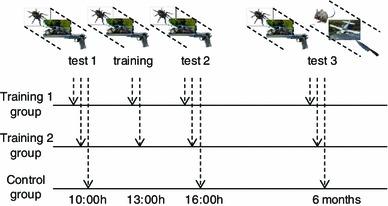
Experimental design

In addition to the above, the participants in all three groups participated in a second part of the experiment, which was performed in the afternoon 6 months after the first part.^3^ This second part was the same for all three groups (see Fig. 4, test 3). In this part, participants were presented the same 5 × 30 pictures from the IAPS picture set as presented in the first part, and were again asked to rate the emotional intensity of the pictures on a scale from 1 to 5. This was done to address the second research question mentioned above, i.e., studying the long-term effects of the different types of training. Next, after a short break, they were again presented 5 × 30 pictures from the IAPS picture set, which were new pictures, but with similar valence and arousal scores as the first 5 × 30 pictures. This was done to find out whether there was any transfer of the training to different pictures, thereby addressing the third research question.

### Implementation

Both the test environment and the two training environments were implemented using the PsychoPy software (http://www.psychopy.org/). This package provides an API for creating psychological experiments using the programming language Python. In combination with the Python API provided by PLUX, all ingredients for implementing both environments were available. The implementation itself is relatively straightforward, looping through the different images in fixed intervals and recording both the physiological measurements from the PLUX device as well as the manual responses from the participants.

## Results

The results of the experiment will be described in three separate sub-sections, addressing the three respective research questions. First, for the first part of the experiment, the two types of training are compared with the control group. Recall that the difference between the three groups was the following:

- Control group: no training
- Training 1 group: choice-reaction task
- Training 2 group: reappraisal task

After this comparison, we investigate whether these types of training have lasting effects, based on the second part of the experiment. Finally, data gathered with a new set of images is compared to the original set to find out if transfer of learning takes place between similar images.

### Type of training

**Fig. 5:**
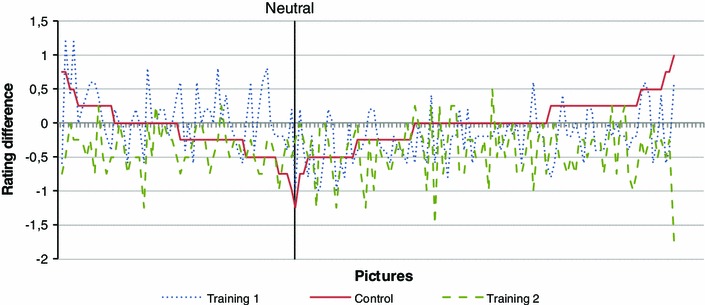
Absolute change in emotional ratings for 150 pictures (averaged over all participants)

Figure 5 shows for all 150 pictures (horizontal axis) the absolute change in emotional ratings (averaged over all participants, vertical axis) between the first and the second test, both for the control group and the two training groups. Pictures are sorted as follows: pictures with a negative valence (i.e., a value smaller than 4.5 according to the IAPS classification) are shown on the left and those with a positive valence on the right hand side. Note that we are particularly interested in the negative images, since emotion regulation training usually aims at decreasing emotional response to negative stimuli. Moreover, both the negative and the positive pictures are sorted with respect to the change in emotional rating of the control group (the solid red line). As can be seen, the curve for the control group is situated around 0 (mean value −0.05), whereas the curves of both training groups are lower (mean value ‘training 1’ −0.11; ‘training 2’ −0.4). A paired *t* test confirmed that training 2 significantly lowered the emotional ratings more than the control group [*t*(149) = −8.15, *p* < 0.001]. However, this change was not significant for the training 1 group [*t*(149) = −1.34, *p* = 0.18]. This indicates that, for this set of participants,^4^ training 2 resulted in significantly lower ratings of the images in the second test.

If we focus on only those images that are negatively valenced (left part of the graph in Fig. 5), the results are different. The mean change for the control group is slightly lower at −0.11. Training 1 resulted in an increase of emotional ratings with a mean change of 0.11, while training 2 still has a mean change of −0.39. Both these changes were confirmed to be significant with *t*(56) = 3.32, *p* = 0.0016 for training 1 and *t*(56) = −4,65, *p* < 0.001 for training 2. From this we conclude that, again for this set of participants, training 1 resulted in a significant increase of emotional ratings for negative images, while training 2 significantly decreased emotional ratings for those images.

**Fig. 6:**
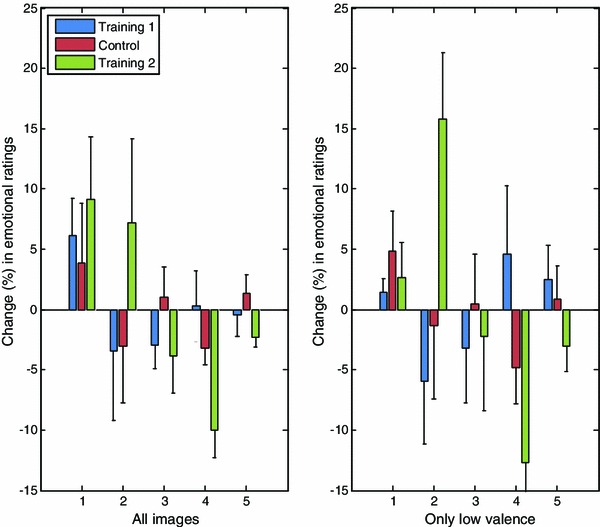
Relative change in emotional ratings

In addition, Fig. 6 shows for both the training groups and the control group, the relative change in emotional ratings between the first and the second round. On the horizontal axis ratings from 1 to 5 are shown. Each bar represents the percentage change between the first and second round for one particular group for that rating. Thus, a positive change represents an increase in the absolute number of pictures that were given that rating in the afternoon.

Looking at the left graph, showing the results for all images, it can be seen that there are small differences between the control group and training 1, in line with the findings above. For training 2, it is clear that there is a decrease in the pictures with a high rating and a corresponding increase of pictures with a low rating.

Focussing on only those images with a negative valence as shown in Fig. 6 on the right, the results for both the control group and training 2 show a similar pattern compared with all images. However, for training 1, a trend can be seen towards the higher emotional ratings. This is in compliance with the results above, where we found a significant increase of the mean ratings for this group.

**Fig. 7:**
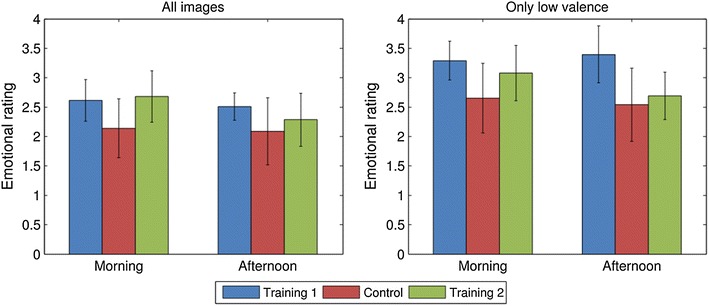
Absolute change in emotional ratings

Finally, in addition to comparing the absolute (Fig. 5) as well as the relative (Fig. 6) change in emotional ratings among the different groups, we explicitly compared the average rating given in the afternoon with those given in the morning for each group individually (Fig. 7). We found that training 2 is the only group in which a significant drop of emotional ratings has occurred for all images [*t*(3) = 7.57, *p* = 0.048] as well as for only the negative images [t(3) = 9.69, *p* = 0.023]. For both the control group and training 1 the differences between the morning and afternoon measurements were not significant. This provides strong evidence for the hypothesis that reappraisal-based virtual training can be used to reduce subjects’ emotional responses to negative stimuli at later times.

### Six months later

**Fig. 8:**
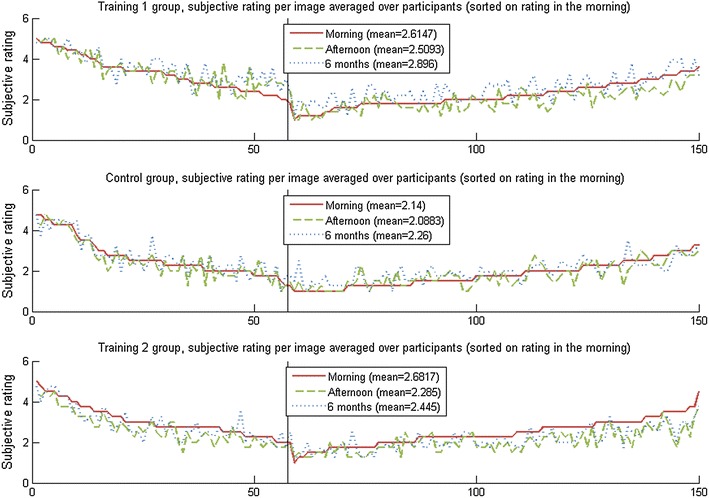
Emotional ratings for 3 different points in time

Figure 8 shows the subjective emotional ratings for the two measurements (test 1 and 2, i.e., morning and afternoon) in the first part of the experiment as well as the same measurement (test 3) using the same participants 6 months later. Pictures are sorted on the emotional rating given during the first measurement, such that those pictures with a negative valence are shown on the left and those with a positive valence on the right hand side. For the control group (middle graph), it can be seen that both later measurements vary around the initial ratings given for those images, and even so no significant differences were found.

For training 1 (top graph) the difference is harder to see. However, statistically, the measurement in the afternoon gave slightly lower ratings overall [*t*(149) = 3.0478, *p* = 0.0027], while 6 months later the ratings had increased compared to the first measurement [*t*(149) = −8.1722, *p* < 0.0001]. Taking into account only the negative images, the mean rating increased from 3.29 in the morning, via 3.40 in the afternoon, to 3.56 after 6 months, with both the difference between the morning and the 6 months as well as that between the afternoon and 6 months measurement being significant [*t*(56) = −4.4742, *p* < 0.0001 and *t*(56) = −2.6364, *p* = 0.011 respectively].

The lower graph in Fig. 8 (training 2) shows that both the measurement taken in the afternoon and the one taken 6 months later resulted on average in lower ratings, whereby the ratings after 6 months roughly lie between the ratings of the other two time points measured. Furthermore, all these differences are statistically significant with *p* < 0.0001. Regarding the negative images, similar results are found with statistical significance of *p* < 0.001.

Thus, after 6 months, the control group still had a similar response towards the images as they had initially. The participants taking part in training 1 already showed an increased response towards the negative images in the afternoon, and after 6 months this had increased even more for the negative images as well as the complete set of images. The lowered emotional response caused by training 2 was still present (and significant) after 6 months, albeit less pronounced.

### Transfer of training

**Fig. 9:**
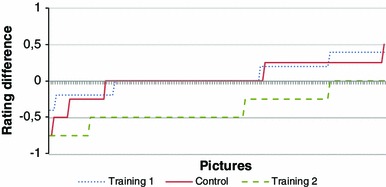
Differences in emotional ratings between two different sets of pictures

Six months after the initial experiment, each participant also rated a second set of images on the emotional intensity. Those images were randomly selected from the remaining IAPS pictures, with the constraint that the set as a whole matched to the first set on both valence and arousal. Figure 9 shows the differences in ratings between the initial measurement of the first set of pictures and the ratings for the new images after 6 months. If transfer of training would take place, or in case of the control group with no training at all, similar results would be expected for both sets of stimuli. This can also be seen in the graph, were the curve for the control group is situated very closely around 0. As expected, for this group no significant differences were found using an unpaired t-test for either all images or only the negative ones [*t*(298) = −0.3462, *p* = 0.73; *t*(113) = −0.0933, *p* = 0.93].

Furthermore, Fig. 9 shows very few differences between the control group and training 1, indicating little transfer regarding this type of training. An unpaired *t* test confirms this: *t*(298) = -0.62703, p = 0.73 for all images and *t*(113) = −1.0934, *p* = 0.14 for the negative ones. For training 2, the mean rating has dropped, which can also been seen in Fig. 9. These differences were significant for all images [*t*(298) = 4.342, *p* < 0.0001] as well as for only the negative pictures [*t*(113) = 1.7808, *p* = 0.039]. Thus, although no transfer can be shown for training 1, transfer does seem to take place for training 2.

## Discussion

In this paper an experiment is reported addressing the impact of virtual training on participants’ experienced emotional responses towards negative stimuli. Participants were asked to rate the subjective emotional intensity of a set of affective pictures at two different points time. The participants were divided into a first group performing a session of virtual training in between these time points, a second group performing virtual training thereby applying reappraisal strategies, and a control group without any training session. The results are that the reappraisal-based training caused the participants in that group to give significantly lower ratings for the emotional intensity of the negative pictures, whereas the content-based training resulted in significantly higher ratings compared to the group without training. Moreover, a second experiment, performed with the same participants 6 months later, indicated that these effects are fairly persistent over time, and that transfer to pictures with similar characteristics takes place.

The outcomes of this experiment indicate that, depending on its setup, virtual training may either strengthen the emotional responses to stimuli or weaken them. The fact that the first group shows enhanced responses might be explained by a form of fear conditioning (e.g., [25]) taking place in this setup. By presenting the stimuli in an active, dynamic manner, and by asking the subjects to perform an action as a response, a state of enhanced attention on the stimuli was induced, which may have an opposite effect compared to, for example, the emotion regulation strategy called attention deployment (cf. [23]), and by a process of fear conditioning this may lead to a form of up-regulation as opposed to down-regulation. In contrast to this, in the second group it was explicitly asked to apply an emotion regulation strategy based on reappraisal (cf. [23]). The outcomes indicate that indeed such a setup can strengthen the emotion regulation, which can be explained as inducing a form of fear extinction learning [26, 27].

**Fig. 10:**
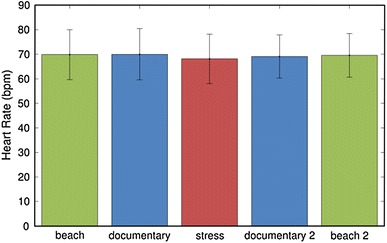
Average heart rate and standard deviation for each video clip (taken from [28] )

**Fig. 11:**
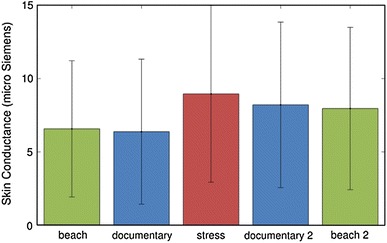
Average heart rate and standard deviation for each video clip (taken from [28] )

As a next step, an exploration has been made regarding the possibilities to induce a stress response through video material. Preliminary results of this exploration are reported in [28]. Here, a pilot experiment has been performed in which participants were asked to watch five different video clips while their emotional response was measured via physiological measurements as well as questionnaires. Among the five movies, the third one was composed in such a way that it could be experienced as being stressful, whereas the clip prior to this movie designed to serve as a neutral movie, to determine the baseline level of heart rate and skin conductance of the participants. The results of the measurements showed that the heart rate of the subjects did not differ significantly during the stressful video clip compared to the other four video clips (see Fig. 10). Instead, the skin conductance of the participants increased significantly during the stressful clip (see Fig. 11), and the same held for the subjective ratings. Hence, we conclude that it is also possible to generate a stress response by means of video material, and that skin conductance is an effective indicator to measure this.

For further research, it is planned to perform more experiments like this, with more participants and a greater focus on interpersonal differences. At the moment, similar experiments are being conducted with different types of stimuli (such as sounds and games [29]) to elicit emotional responses. An interesting additional element here is a personality questionnaire to consider individual differences in relation to for example specific personality traits. This opens up the possibility to investigate whether particular personality traits indicate what type of training would be most beneficial for that particular person.

Finally, the aim of the project is to build a VR training environment in which the knowledge acquired is incorporated. In this respect, care should be taken not to over-generalise the results of the current study. It is obvious that there is still a large gap in realism between scary images or video clips on the one hand, and confrontations with real-world threatening stimuli on the other hand. As this gap cannot be closed by means of one single study, the STRESS project takes an incremental approach, where we gradually try to increase realism of the presented stimuli. For instance, in a study that is currently in progress, an experiment is performed in which participants are actually confronted with virtual reality-based stimuli. More specifically, they are interacting with a virtual character, which at some point starts behaving aggressively towards the participant. In addition, another group of participants is being confronted with a real human (an actress), who starts behaving aggressively as well, in the exact same manner as the virtual agent does. Initial results of this experiment are promising: they indicate that in both conditions, the threatening event invokes subjective and physiological responses in the participants, although there are still some subtle differences between the virtual and the real stimuli. In follow-up research, the nature of these differences, as well as their implications for training purposes, will be studied in more detail.

## Footnotes

1 Note that this intensity was independent of the ‘valence’ of the picture (i.e., pos. vs. neg).

2 The results of these measurements are not further discussed in this paper. They were collected to gain more insight in the relation between the presented stimuli and physiological states, which will be further explored in a follow-up experiment.

3 In both the control and training 2 group one participant dropped out.

4 This test took, for each of the 150 pictures, the average change in rating given by the participants in the training group, and compared this with the average change in rating given to the same picture by the participants in the control group. Since this way of testing takes the pictures (instead of the participants) as a basis, the results cannot be generalised for the population as a whole. However, an additional (unpaired two sample) *t*-test has been performed in which for each participant the average change in rating over all 150 pictures was calculated, and these averages were used to compare the training 2 with the control group. Due to the low number of participants, these results were not statistically significant on the p < 0.05 level, but a clear trend was found in the results (with t(8) = 2.05, p = 0.07).
